# Immuno-PET and Targeted α-Therapy Using Anti–Glypican-1 Antibody Labeled with ^89^Zr or ^211^At: A Theranostic Approach for Pancreatic Ductal Adenocarcinoma

**DOI:** 10.2967/jnumed.123.266313

**Published:** 2023-12

**Authors:** Tadashi Watabe, Kazuya Kabayama, Sadahiro Naka, Ryuku Yamamoto, Kazuko Kaneda, Satoshi Serada, Kazuhiro Ooe, Atsushi Toyoshima, Yang Wang, Hiromitsu Haba, Kenta Kurimoto, Takanori Kobayashi, Eku Shimosegawa, Noriyuki Tomiyama, Koichi Fukase, Tetsuji Naka

**Affiliations:** 1 Department of Nuclear Medicine and Tracer Kinetics, Graduate School of Medicine, Osaka University, Suita, Japan;; 2 Institute for Radiation Sciences, Osaka University, Suita, Japan;; 3 Department of Chemistry, Graduate School of Science, Osaka University, Toyonaka, Japan;; 4 Forefront Research Center, Graduate School of Science, Osaka University, Toyonaka, Japan;; 5 Department of Pharmacy, Osaka University Hospital, Suita, Japan;; 6 Institute for Biomedical Sciences Molecular Pathophysiology, Iwate Medical University, Yahaba, Japan;; 7 Nishina Center for Accelerator-Based Science, RIKEN, Saitama, Japan;; 8 Department of Molecular Imaging in Medicine, Graduate School of Medicine, Osaka University, Suita, Japan;; 9 Department of Radiology, Graduate School of Medicine, Osaka University, Suita, Japan; and; 10 Division of Allergy and Rheumatology, Department of Internal Medicine, School of Medicine, Iwate Medical University, Yahaba, Japan

**Keywords:** theranostics, glypican-1, immuno-PET, targeted α-therapy, pancreatic ductal adenocarcinoma, astatine, zirconium

## Abstract

Glypican-1 (GPC1) is overexpressed in several solid cancers and is associated with tumor progression, whereas its expression is low in normal tissues. This study aimed to evaluate the potential of an anti-GPC1 monoclonal antibody (GPC1 mAb) labeled with ^89^Zr or ^211^At as a theranostic target in pancreatic ductal adenocarcinoma. **Methods:** GPC1 mAb clone 01a033 was labeled with ^89^Zr or ^211^At with a deferoxamine or decaborane linker, respectively. The internalization ability of GPC1 mAb was evaluated by fluorescence conjugation using a confocal microscope. PANC-1 xenograft mice (*n* = 6) were intravenously administered [^89^Zr]GPC1 mAb (0.91 ± 0.10 MBq), and PET/CT scanning was performed for 7 d. Uptake specificity was confirmed through a comparative study using GPC1-positive (BxPC-3) and GPC1-negative (BxPC-3 GPC1-knockout) xenografts (each *n* = 3) and a blocking study. DNA double-strand breaks were evaluated using the γH2AX antibody. The antitumor effect was evaluated by administering [^211^At]GPC1 mAb (∼100 kBq) to PANC-1 xenograft mice (*n* = 10). **Results:** GPC1 mAb clone 01a033 showed increased internalization ratios over time. One day after administration, a high accumulation of [^89^Zr]GPC1 mAb was observed in the PANC-1 xenograft (SUV_max_, 3.85 ± 0.10), which gradually decreased until day 7 (SUV_max_, 2.16 ± 0.30). The uptake in the BxPC-3 xenograft was significantly higher than in the BxPC-3 GPC1-knockout xenograft (SUV_max_, 4.66 ± 0.40 and 2.36 ± 0.36, respectively; *P* = 0.05). The uptake was significantly inhibited in the blocking group compared with the nonblocking group (percentage injected dose per gram, 7.3 ± 1.3 and 12.4 ± 3.0, respectively; *P* = 0.05). DNA double-strand breaks were observed by adding 150 kBq of [^211^At]GPC1 and were significantly suppressed by the internalization inhibitor (dynasore), suggesting a substantial contribution of the internalization ability to the antitumor effect. Tumor growth suppression was observed in PANC-1 mice after the administration of [^211^At]GPC1 mAb. Internalization inhibitors (prochlorperazine) significantly inhibited the therapeutic effect of [^211^At]GPC1 mAb, suggesting an essential role in targeted α-therapy. **Conclusion:** [^89^Zr]GPC1 mAb PET showed high tumoral uptake in the early phase after administration, and targeted α-therapy using [^211^At]GPC1 mAb showed tumor growth suppression. GPC1 is a promising target for future applications for the precise diagnosis of pancreatic ductal adenocarcinoma and GPC1-targeted theranostics.

## Visual Abstract

**Figure:**
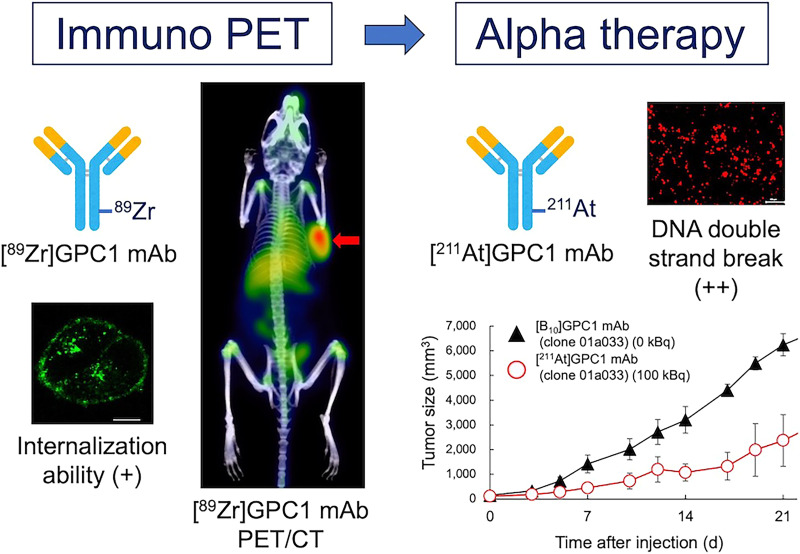

Pancreatic ductal adenocarcinoma (PDAC) is one of the most refractory cancers worldwide. Despite the development of many anticancer drugs, the 5-y survival rate remains lower than 10% (1). Detection of PDAC is sometimes difficult because abnormalities are not shown in CT images for small tumors (2). In addition, some PDACs are not [^18^F]FDG-avid and cannot be detected using conventional [^18^F]FDG PET (3). Therefore, new precise imaging techniques for the early detection of PDAC, as well as the development of new therapies in combination with theranostic approaches, are needed.

Glypicans are a family of heparan sulfate proteoglycans that play diverse roles in growth factor signaling, cell adhesion, and differentiation (4). Among the 6 known glypicans, glypican-1 (GPC1) has gained significant attention because of its aberrant expression in several types of cancer, including pancreatic, breast, and uterine cervical cancers, as well as esophageal squamous cell carcinoma and cholangiocarcinoma (5–9). GPC1 has also been implicated in cancer cell proliferation, invasion, and metastasis, but its expression is low in normal human tissues (10). High expression of GPC1 is reportedly a poor prognostic factor in esophageal cancer, pancreatic cancer, cholangiocarcinoma, and glioblastoma (7,8,11,12). In pancreatic cancer, GPC1 expression is observed not only in cancer cells but also in cancer-associated fibroblasts of the stroma (13). As a potential therapeutic target, an anti-GPC1 monoclonal antibody (GPC1 mAb) drug conjugate has been developed and has shown excellent preclinical in vivo antitumor effects in pancreatic cancer models (7,13). Therefore, GPC1 is a promising target for cancer diagnosis and treatment.

Immuno-PET has emerged as a promising noninvasive imaging modality that uses radiolabeled antibodies (14). It allows targeted detection and quantification of specific molecular targets expressed in cancer cells with high sensitivity and specificity. Among the various radioisotopes available for immuno-PET, ^89^Zr has gained significant attention because of its favorable properties, such as its long physical half-life (78.4 h), labeling capability with deferoxamine, and derivative chelators (15). ^89^Zr-labeled antibodies have been investigated in preclinical and clinical studies for imaging various types of cancer (15). However, their availability for PDACs remains limited, possibly because of the abundant stroma in PDAC, which prevents the delivery of the antibody to the target; thus, there is a need for the development of new ^89^Zr-labeled antibodies with higher sensitivity (16).

In recent years, emphasis has been placed not only on diagnostic imaging but also on the development of radionuclide therapy as a theranostic agent. Targeted α-therapy is gaining increasing attention, because it can deliver greater therapeutic efficacy than can conventional β-emitters (17). Among α-emitting radionuclides, ^211^At is attracting attention because it can be produced by irradiating natural bismuth targets, which are abundant in nature, with α-beams using a 30-MeV cyclotron (18). In this study, the potential of a GPC1 mAb labeled with ^89^Zr or ^211^At as a theranostic target for PDACs was evaluated using a xenograft model.

## MATERIALS AND METHODS

### Synthesis of [^89^Zr]GPC1 mAb

^89^Zr was produced via the nuclear reaction of ^89^Y(p, n)^89^Zr using a medical cyclotron, Cypris HM-18 (Sumitomo Heavy Industries). Mouse GPC1 mAb clone01a033 and GPC1 mAb clone T2 (a humanized antibody of clone 01a033) were generated as previously described (5,9). [^89^Zr]GPC1 mAb was prepared by labeling deferoxamine-linked GPC1 mAb with ^89^Zr according to previously reported methods (19). GPC1 mAb (2–2.5 mg) was dissolved in 1 mL of 0.1 M sodium bicarbonate buffer (pH 9.0) and reacted with 4–6.5 μL of 10 mM *p*-SCN-Bn-deferoxamine (Macrocyclics) in dimethyl sulfoxide using ThermoMixer C (Eppendorf) for 30 min (at 37°C and 550 rpm). The GPC1 mAb conjugate with deferoxamine (2 mL of solution) was purified from the reaction solution using a PD-10 column (Cytiva) and 5 mg/mL gentisic acid in 0.25 M sodium acetate buffer (pH 5.5). Next, 0.05 mL of 2 M Na_2_CO_3_ was added to 0.3 mL of ^89^Zr solution (70–124 MBq) and incubated for 3 min. Thereafter, 0.2 mL of 0.5 M *N*-(2-hydroxyethyl)piperazine-*N*′-(2-ethanesulfonic acid) buffer (pH 7.2) and 0.6 mL of GPC1 mAb conjugate with deferoxamine solution were added and the pH was adjusted to 6.8 using 2 M Na_2_CO_3_, followed by a reaction at 27°C and 550 rpm for 60 min. [^89^Zr]GPC1 mAb was obtained with a radiochemical yield of 17% on average and more than 90% radiochemical purity via radio–thin-layer chromatography analysis by purifying the reaction solution using a PD-10 column and 5 mg/mL gentisic acid in 0.25 M sodium acetate buffer (pH 5.5).

### Synthesis of [^211^At]GPC1 mAb

^211^At was produced via the nuclear reaction of ^209^Bi(α, 2n)^211^At using azimuthally varying field cyclotrons (Research Center for Nuclear Physics, Osaka University, and Radioactive Isotope Beam Factory, Institute of Physical and Chemical Research) and purified using a dry distillation method (17). The isolated ^211^At was introduced into previously prepared [B]_10_-GPC1 mAbs (clone 01a033 or clone 1-12). The synthesis of [B]_10_-GPC1 mAb is described in the supplemental materials (supplemental materials are available at http://jnm.snmjournals.org). The ^211^At solution (clone 01a033, 13.3 MBq; clone 1-12, 12.0 MBq) and phosphate-buffered saline were added to a solution of chloramine-T in distilled water (0.5 mg/mL). Next, [B]_10_-GPC1 mAbs (40 μg each) in phosphate-buffered saline were added to this solution and incubated at room temperature for 10 min. This reaction was quenched using Na_2_S_2_O_5_ (0.5 mg/mL in 6 μL of distilled water). The solution was then transferred to a filter column (Cosmospin Filter H; Nacalai Tesque) packed with chromatographic resin (Sephadex G-25 DNA grade; Cytiva). The solution was centrifuged at 1,300 *g* for 2 min at room temperature to obtain [^211^At]GPC1 mAb. Radiation was measured using a germanium semiconductor detector (BE2020; Mirion Technologies). The radiochemical yields of [^211^At]GPC1 mAbs were calculated at 59.8% and 45.7%, respectively. The purity of [^211^At]GPC1 mAb clone 01a033 and [^211^At]GPC1 mAb clone 1-12 was checked via thin-layer chromatography (with methanol as the eluent) using a radioimager (Typhoon FLA 7000; Cytiva), and the radiochemical purity was more than 90%. An average of 2.3 decaborates was estimated to be bound to GPC1 mAb using matrix-assisted laser desorption/ionization time of flight mass spectrometry.

### Evaluation of the Internalization Ability of GPC1 mAb

An internalization assay was performed using GPC1 mAbs with fluorescence conjugation in PANC-1 cells in the presence of dynasore. Dynasore is a drug that noncompetitively inhibits the guanosine triphosphatase activity of Dynamin1, a guanosine triphosphatase protein responsible for membrane fission during endocytosis. Detailed methods are described in the supplemental materials.

### Preparation of Xenograft Models

PANC-1 and GPC1-positive (BxPC-3) cells were obtained from the American Type Culture Collection and European Collection of Authenticated Cell Cultures, respectively. GPC1-negative (BxPC-3 GPC1-knockout) cells were established as previously described (6). The cells were maintained in culture medium (RPMI 1640 medium with l-glutamine and phenol red [Fujifilm Wako Pure Chemical] for both PANC-1 and BxPC-3 or BxPC-3 GPC1-knockout cells) with 10% heat-inactivated fetal bovine serum and 1% penicillin–streptomycin.

Male nude mice and female nonobese diabetic/severe combined immunodeficiency mice were purchased from Japan SLC Inc. and Charles River Japan Inc., respectively. Tumor xenograft models were established by subcutaneous injection of PANC-1 (1 × 10^7^ cells in nude mice) or BxPC-3 (5 × 10^6^ cells in nonobese diabetic/severe combined immunodeficiency mice) suspended in phosphate-buffered saline (0.1 mL) and Matrigel (1:1; BD Biosciences). The mice were assessed 3 wk after implantation when the tumor size reached approximately 1 cm in diameter. The protocol was approved by the Animal Care and Use Committee of the Osaka University Graduate School of Medicine (approval number 30-088-009). Euthanasia was performed under deep anesthesia by isoflurane inhalation when signs of intolerable suffering or a significant decrease in body weight were observed.

### [^89^Zr]GPC1 mAb PET Imaging and Analysis

[^89^Zr]GPC1 mAb (0.91 ± 0.10 MBq, ∼27 ± 3.0 μg of mouse GPC1 mAb, clone 01a033) was intravenously administered to PANC-1 xenograft mice (9 wk old; body weight, 23.4 ± 0.9 g; *n* = 6). PET/CT scanning was performed 1 h, 1 d, 2 d, 4 d, and 7 d after administration under isoflurane anesthesia using a small-animal PET scanner (Siemens Inveon PET/CT). After the PET scan, the mice were euthanized, and the radioactivity and weight of the major organs were determined using a well counter (BeWell; Molecular Imaging Laboratory). A comparison of tumoral uptake between GPC1-positive and GPC1-negative xenografts was performed to confirm the specificity of [^89^Zr]GPC1 mAb accumulation for GPC1. [^89^Zr]GPC1 mAb (1.34 ± 0.14 MBq, 34 ± 3.6 μg of humanized GPC1 mAb, clone T2) was intravenously administered to BxPC-3 and BxPC-3 GPC1-knockout xenograft mice (9 wk old; body weight, 17.3 ± 1.6 g; each *n* = 3). PET/CT scanning was performed 24 h after the administration of [^89^Zr]GPC1 mAb, followed by the measurement of biodistribution. The blocking study was also performed by injection of a nonradiolabeled GPC1 mAb (300 μg) before injection of [^89^Zr]GPC1 mAb (0.3–34 μg of humanized GPC1 mAb, clone T2). Biodistribution was compared among the following 4 groups of the PANC-1 xenograft model (each *n* = 3) 24 h after the administration of [^89^Zr]GPC1 mAb: 20 kBq (24 ± 3 kBq, 0.36 ± 0.04 μg), 200 kBq (199 ± 14 kBq, 3.01 ± 0.21 μg), 2 MBq (2.29 ± 0.05 MBq, 34.8 ± 0.78 μg), and 2 MBq with blocking (2.26 ± 0.05 MBq, 34.2 ± 0.79 μg). PET/CT scanning was performed 24 h after the administration of [^89^Zr]GPC1 mAb except in the 20-kBq group.

All PET data were reconstructed using 3-dimensional ordered-subset expectation maximization (16 subsets and 2 iterations), followed by maximum a posteriori estimation with scatter and attenuation correction. The regional uptake of radioactivity was decay-corrected to the injection time and expressed as SUV. Ellipsoid sphere regions of interest were manually placed on the tumor, heart, lungs, liver, kidneys, spleen, and bone joints using PMOD (version 3.6; PMOD Technologies).

### Targeted α-Therapy Using [^211^At]GPC1 mAb

DNA double-strand breaks were evaluated using the γH2AX antibody as previously described (20). PANC-1 cells were seeded in 35-mm glass-based dishes and treated with 100 μM dynasore for 30 min or left untreated. [^211^At]GPC1 mAb (149.58 kBq) was added to these cells, which were then incubated for 120 min. After washing, Hoechst 33342 was added, and the cells were incubated for 10 min. The cells were washed and fixed with 4% paraformaldehyde phosphate buffer and then permeabilized and antibody-stained with anti-γH2AX antibody conjugated with Alexa Fluor 594 (ab206898; Abcam). A fluorescence microscope (BZ-X810; Keyence) with a 20× lens (CFI S Plan Fluor 20XC, numeric aperture 0.45; Nikon) was used for imaging.

PANC-1 xenograft mice (10 wk old; body weight, 18.4 ± 1.9 g; *n* = 20) were divided into 4 groups receiving intravenous administration of [^211^At]GPC1 mAb clone 01a033 (102.80 ± 9.87 kBq, 1.94 ± 0.19 μg, *n* = 5), nonradiolabeled GPC1 mAb clone 01a033 (2.08 ± 0.17 μg, *n* = 5), [^211^At]GPC1 mAb clone 1-12 (105.20 ± 12.19 kBq, 1.72 ± 0.20 μg, *n* = 5), and nonradiolabeled GPC1 mAb clone 1-12 (1.98 ± 0.21 μg, *n* = 5). Tumor size (mm^3^) and body weight (g) were monitored to evaluate treatment effects and systemic side effects. The therapeutic effect of internalization inhibition was also evaluated by administering the endocytosis inhibitor prochlorperazine (21). Detailed methods for immunohistochemistry are described in the supplemental materials.

### Statistical Analyses

Comparisons between the 2 groups were performed using the Mann–Whitney *U* test or *t* test in SPSS (version 25.0; IBM Corp.). Differences were considered statistically significant if the *P* value was less than 0.05.

## RESULTS

**FIGURE 1.**
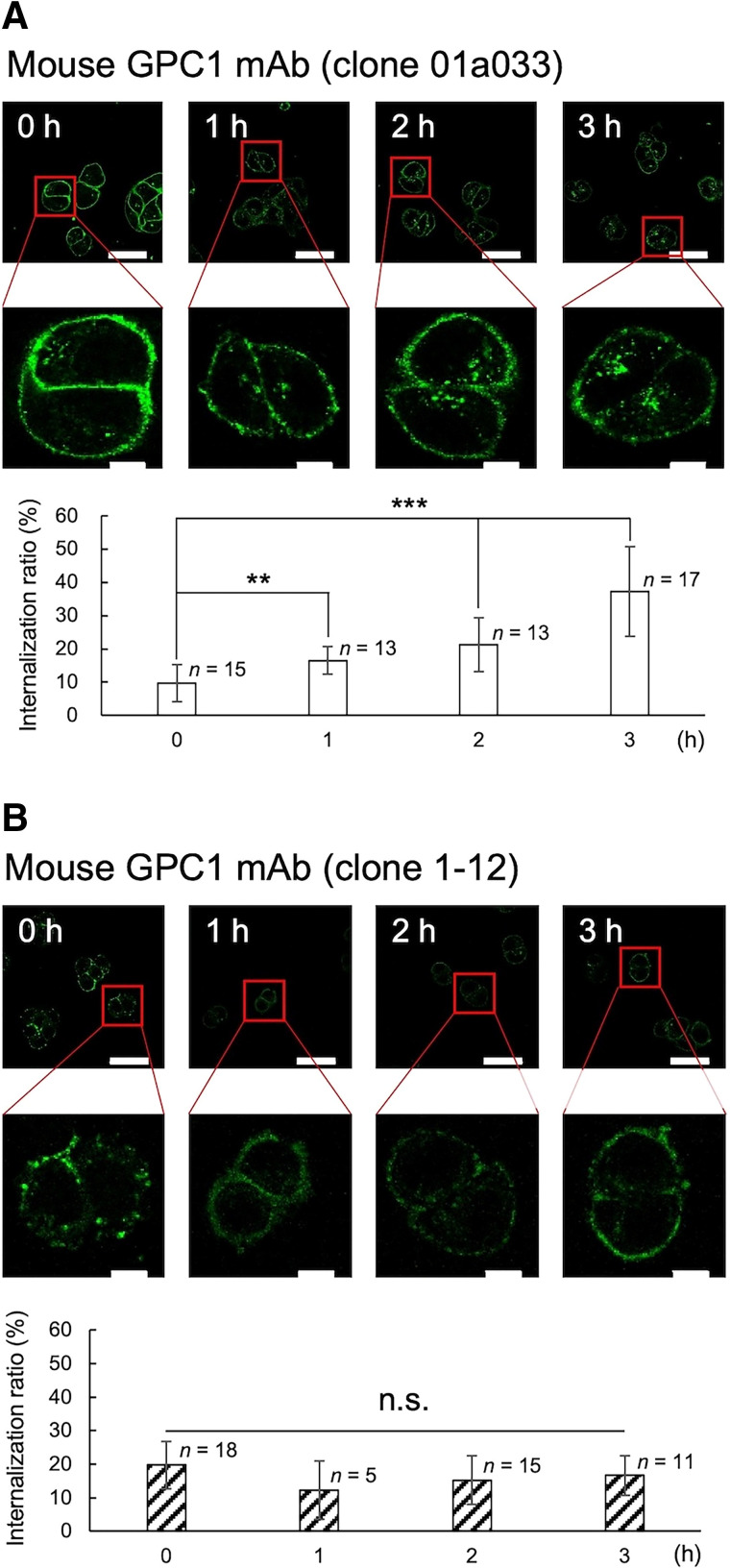
Evaluation of internalization ability in PANC-1 cells using imaging analysis. Changes in internalization ratios of mouse GPC1 mAb are shown for clone 01a033 (A) and clone 1-12 (B). White scale bars indicate 50 μm in upper row and 10 μm in lower row. Graph shows average intracellular internalization rates, with error bars indicating SD. **P < 0.01. ***P < 0.001. ns = not significant.

**FIGURE 2.**
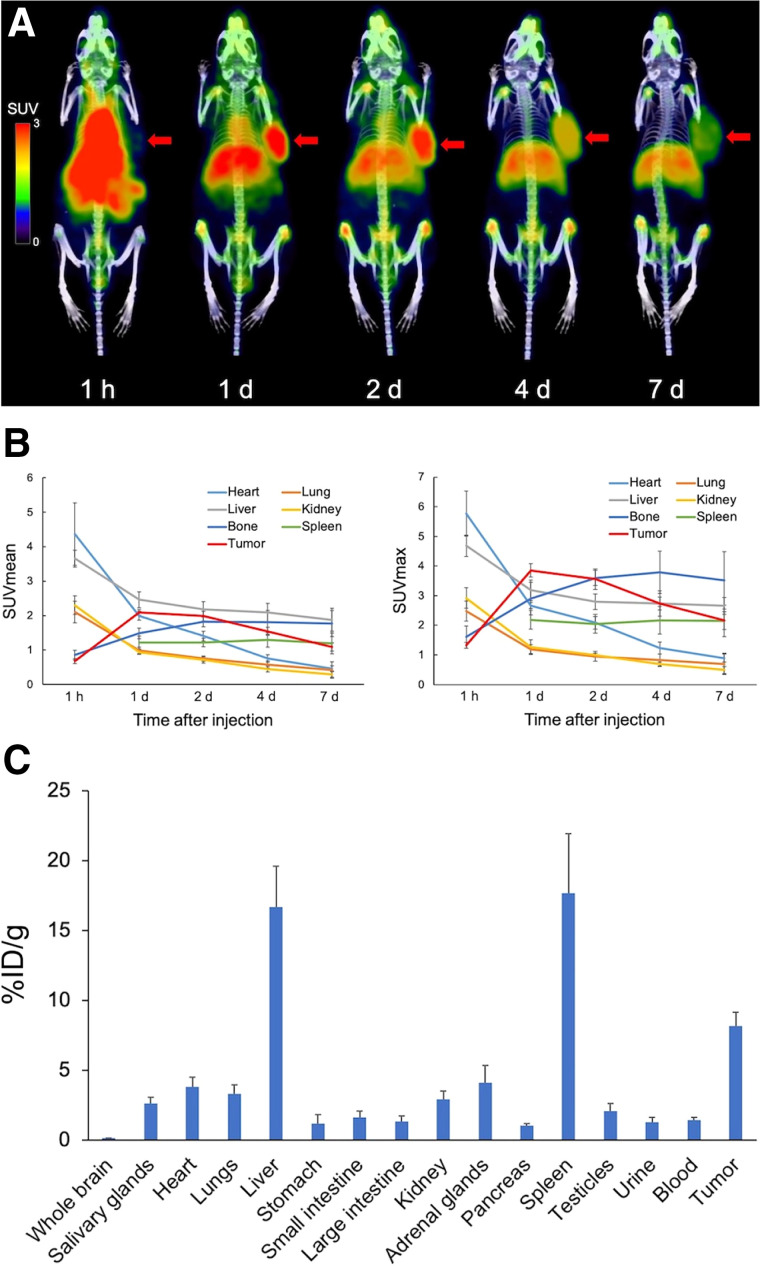
(A) [^89^Zr]GPC1 mAb PET images (clone 01a033) in PANC-1 xenograft mice (arrows indicate PANC-1 xenografts). (B) Quantitative analyses of [^89^Zr]GPC1 mAb PET (clone 01a033) in PANC-1 xenograft mice. Uptake in spleen at 1 h could not be evaluated because of spillover from high uptake in liver. (C) Biodistribution of [^89^Zr]GPC1 PET (clone 01a033) in PANC-1 xenograft mice (day 7).

The internalization ability of GPC1 mAb is shown in Figure 1 and Supplemental Figure 1. GPC1 mAb clone 01a033 showed increased internalization ratios over time, whereas GPC1 mAb clone 1-12 showed no significant internalization (Fig. 1). A significant blocking effect of dynasore on internalization was observed for GPC1 mAb clone 01a033 (Supplemental Figs. 1A–1C). Antibodies with high internalization abilities are suitable for targeted α-therapy because of their increased probability of interacting with DNA in the nucleus. Therefore, clone 01a033 was used for subsequent PET and α-therapy experiments. PET images of PANC-1 xenograft mice after the administration of [^89^Zr]GPC1 mAb clone 01a033 are shown in Figure 2A. A high accumulation of [^89^Zr]GPC1 mAb was observed in the tumor xenograft 1 d after administration (SUV_max_, 3.85 ± 0.10), and this level then gradually decreased until day 7 (SUV_max_, 2.16 ± 0.30; Figs. 2A and 2B). Relatively high accumulation was observed in the liver, spleen, and bone (epiphysis) and was considered physiologic accumulation of GPC1 mAb or ^89^Zr. The whole-body distribution of [^89^Zr]GPC1 mAb clone 01a033 is shown in Figure 2C. High uptake was observed in the liver (16.7 ± 2.9 percentage injected dose [%ID]/g), spleen (17.7 ± 4.3 %ID/g), and PANC-1 tumor (8.2 ± 1.0 %ID/g), suggesting that the liver and spleen could be at-risk organs considering the residence time. Accumulation in the spleen was considered physiologic accumulation of IgG rather than GPC1-mediated accumulation.

**FIGURE 3.**
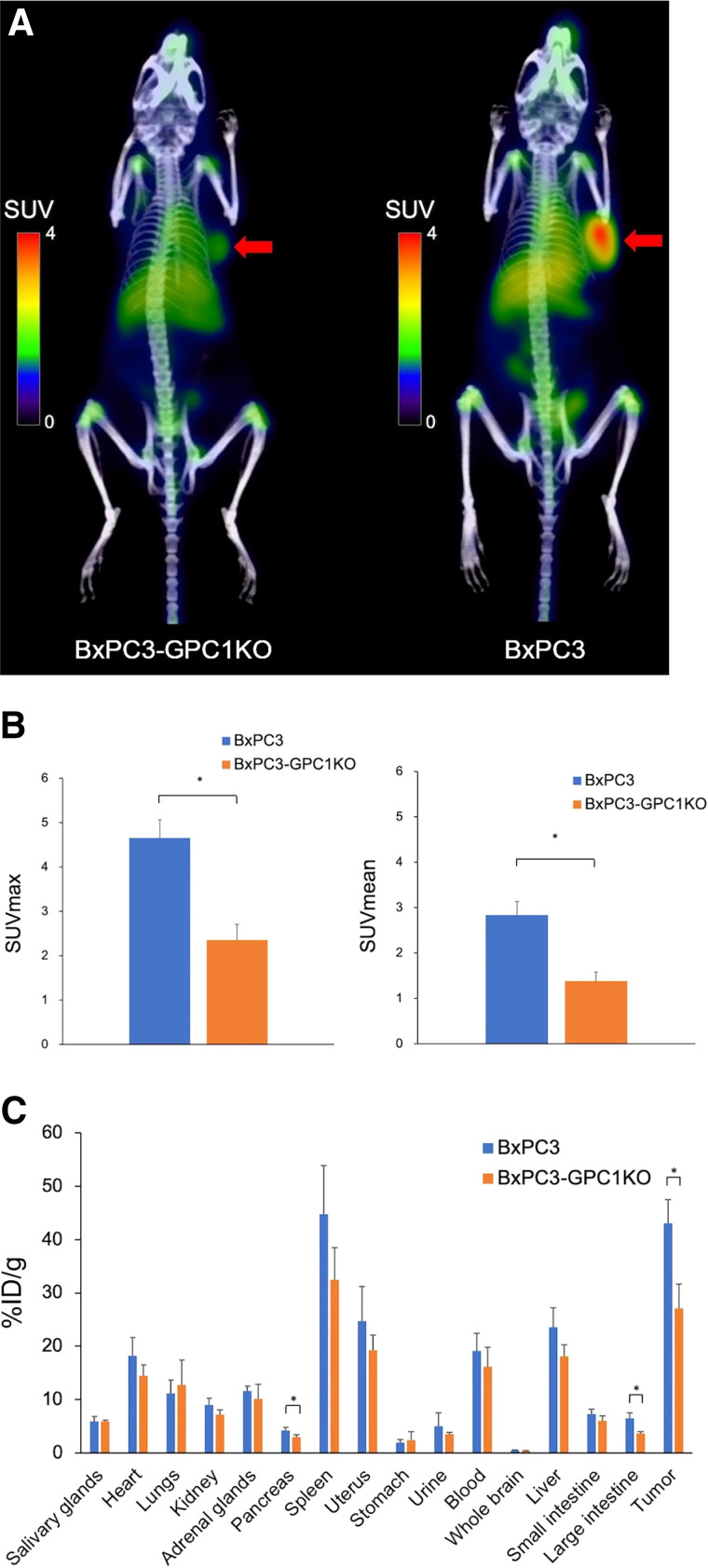
(A) [^89^Zr]GPC1 mAb PET images (clone T2) in BxPC-3 and BxPC-3 GPC1-knockout xenograft mice (arrows indicate tumor xenografts). (B) Quantitative analyses of tumoral uptake on [^89^Zr]GPC1 mAb PET (clone T2). (C) Biodistribution of [^89^Zr]GPC1 mAb clone T2 24 h after administration. *P = 0.05 using Mann–Whitney *U* test. KO = knockout.

To evaluate the specificity, tumoral uptake was compared between BxPC-3 and BxPC-3 GPC1-knockout tumor models 24 h after the administration of [^89^Zr]GPC1 mAb clone T2. The uptake in the BxPC-3 xenograft was significantly higher than in the BxPC-3 GPC1-knockout xenograft (SUV_max_, 4.66 ± 0.40 and 2.36 ± 0.36, respectively; *P* = 0.05; Figs. 3A and 3B). Regarding biodistribution, the BxPC-3 xenograft exhibited significantly higher uptake than did the BxPC-3 GPC1-knockout xenograft (43.1 ± 4.5 and 27.1 ± 4.5 %ID/g, respectively; *P* = 0.05), whereas a similar distribution was observed in the normal organs between the 2 groups (Fig. 3C).

**FIGURE 4.**
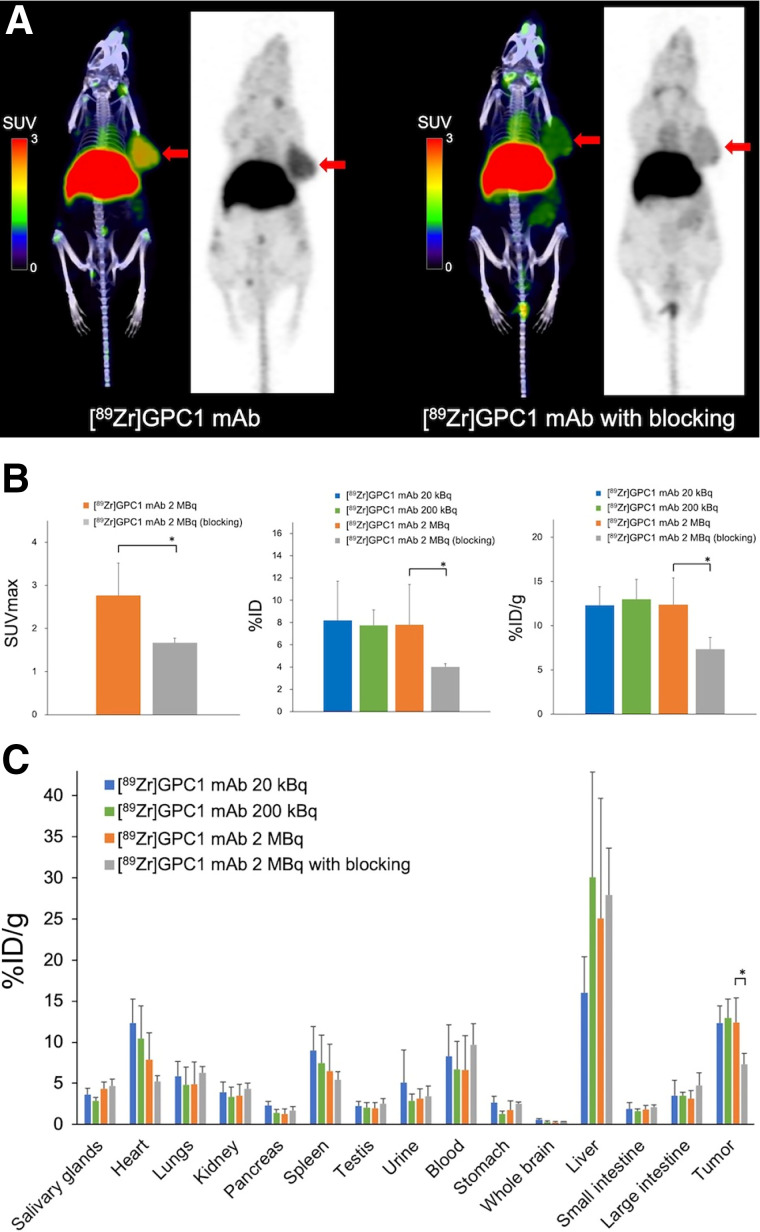
(A) Comparison of [^89^Zr]GPC1 mAb PET/CT images (clone T2) with and without injection of nonradiolabeled GPC1 mAb (300 μg) before injection of [^89^Zr]GPC1 mAb clone T2 in PANC-1 xenograft mice (arrows indicate PANC-1 xenografts). (B) Comparison of tumoral uptake on [^89^Zr]GPC1 mAb PET (clone T2) and biodistribution. (C) Whole-body distribution of [^89^Zr]GPC1 mAb clone T2 24 h after administration. *P = 0.05 compared with blocking group.

Biodistribution was compared 24 h after the administration of [^89^Zr]GPC1 mAb clone T2 among the 4 PANC-1 xenograft model groups: 20 kBq, 200 kBq, 2 MBq, and 2 MBq with blocking. [^89^Zr]GPC1 mAb PET showed a significant decrease in the blocking group (Fig. 4A). The uptake was significantly inhibited in the blocking group compared with the nonblocking group (7.3 ± 1.3 and 12.4 ± 3.0 %ID/g, respectively; *P* = 0.05; Figs. 4B and 4C). In the heart and spleen, there was a decreasing trend as the amount of antibody increased (Fig. 4C).

**FIGURE 5.**
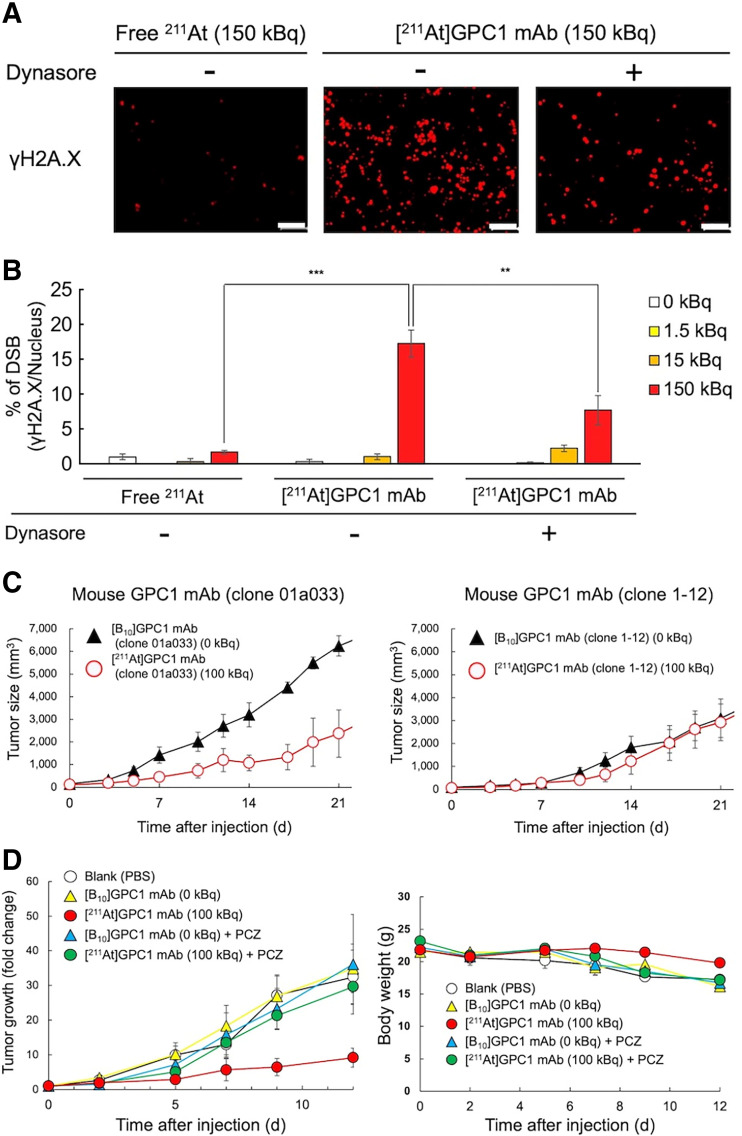
(A and B) Evaluation of DNA double-strand breaks with and without inhibitors (dynasore). White scale bars indicate 100 μm, *n* = 3. **P < 0.01. ***P < 0.001. (C) Tumor growth curves after administration of [^211^At]GPC1 mAb or nonradiolabeled mAb (left, clone 01a033; right, clone 1-12). (D) Tumor growth curves and changes in body weight after administration of [^211^At]GPC1 mAb or nonradiolabeled mAb clone 01a033 with or without administering endocytosis inhibitor. PCZ = prochlorperazine.

DNA double-strand breaks were observed in PANC-1 cells by adding 150 kBq of [^211^At]GPC1 mAb (Figs. 5A and 5B). Double-strand break induction was significantly suppressed by the internalization inhibitor (dynasore), suggesting a substantial contribution of the internalization ability to the antitumor effect. Tumor growth suppression was observed in PANC-1 xenograft mice after the administration of [^211^At]GPC1 mAb clone 01a033 (antibodies with internalization ability) compared with nonradiolabeled GPC1 mAb clone 01a033 (Fig. 5C). However, [^211^At]GPC1 mAb clone 1-12 (antibodies without internalization ability) showed no treatment effect compared with the nonradiolabeled GPC1 mAb clone 1-12. Internalization inhibitors (prochlorperazine) significantly inhibited the therapeutic effect of [^211^At]GPC1 mAb clone 01a033 (Fig. 5D). There was no significant change in body weight after the administration of each mAb (Fig. 5D).

**FIGURE 6.**
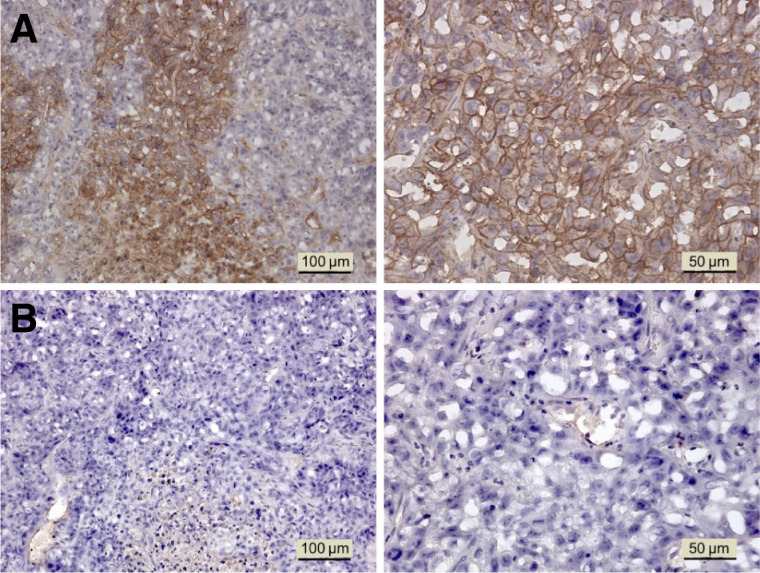
(A) GPC1 immunostaining in PANC-1 xenograft (left, low magnification, ×200; right, high magnification, ×400). (B) Negative controls of corresponding sections without adding primary GPC1 mAb.

Immunohistochemical staining showed high GPC1 expression in the PANC-1 xenograft (Fig. 6). In the normal organs, GPC1 was only weakly positive in the liver, adrenal glands, and testes (Supplemental Fig. 2).

## DISCUSSION

In this study, the whole-body distribution of [^89^Zr]GPC1 mAb was evaluated using a xenograft model, and the therapeutic effect of [^211^At]GPC1 mAb with internalization ability was assessed. [^89^Zr]GPC1 mAb demonstrated high tumoral uptake via PET as early as day 1, which gradually decreased until day 7. Through a comparative study using GPC1-negative tumors and a blocking study, the in vivo specificity of [^89^Zr]GPC1 mAb against GPC1 was confirmed, which was compatible with the high expression of GPC1 in the PANC-1 xenografts.

Immuno-PET using full antibodies labeled with ^89^Zr typically requires a long time to reach maximum uptake. Previous studies using [^89^Zr]trastuzumab demonstrated that it took more than 3–5 d to achieve maximum uptake on PET in both xenograft models and patients with cancer (22–23). Considering the physical half-life of ^211^At (7.2 h), it is more suitable than ^225^Ac for the labeling of GPC1 mAb, which has a longer half-life of 10 d. In addition, the low expression of GPC1 in normal organs is a favorable characteristic for targeted α-therapy to reduce the side effects caused by off-target accumulation.

The GPC1 mAb used in this study possesses an internalization ability, which may contribute to its high uptake in the early phase. Moreover, this study demonstrated that the internalization ability is essential for inducing therapeutic effects in the targeted α-therapy of [^211^At]GPC1 mAb (Fig. 5). Given that the range of α-radiation is as short as 100 μM, it is more efficient to irradiate from the inside of the cell to increase the likelihood of targeting the DNA within the nucleus.

[^18^F]FDG PET is commonly used for staging and detecting recurrence in patients with PDAC. However, in some PDACs, [^18^F]FDG PET may show only faint or no abnormal uptake at the primary site or in metastatic lesions (3). [^18^F]FAPI-74 PET is more useful than [^18^F]FDG PET in detecting the metastatic lesion in PDAC, but caution is required, because it also shows high accumulation in pancreatitis (3). Although further translational research is needed to prove its clinical potential, [^89^Zr]GPC1 mAb PET may be used for the precise staging or early detection of PDAC. Furthermore, GPC1-targeted mAb drug conjugates have been developed and have shown great potential for the effective treatment of refractory PDAC and esophageal cancer (5,11,13). [^89^Zr]GPC1 mAb may serve as a companion diagnostic modality for selecting suitable patients before administering a therapeutic GPC1 mAb drug conjugate. Although we previously detected the expression of GPC1 in the liver metastasis of PDAC (5), the expression levels of the target molecule may exhibit heterogeneity among individual metastatic lesions within the same patient. Therefore, whole-body evaluation using [^89^Zr]GPC1 mAb PET is an ideal method compared with biopsy from a single lesion or blood biomarker, because the former can assess only a portion of 1 lesion and the latter can provide averaged information irrespective of the overall tumor burden.

In a comparison between a BxPC-3 and a BxPC-3 GPC1-knockout tumor, mild accumulation was observed 24 h after the administration of [^89^Zr]GPC1 mAb. This can be attributed to the enhanced permeability and retention effect, wherein antibodies tend to accumulate nonspecifically in tumor regions with increased vascular permeability. In addition, it is possible that the accumulation of [^89^Zr]GPC1 mAb was observed in mouse-derived, cancer-associated fibroblasts present in the stroma of BxPC-3 GPC1-knockout tumors because of the expression of GPC1 (Supplemental Fig. 3). The uptake of [^89^Zr]GPC1 mAb PET was more than 2-fold higher in the BxPC-3 xenograft than in the BxPC-3 GPC1-knockout model, suggesting that specific uptake is mediated by GPC1.

^211^At is a promising α-emitter because of its production capability using natural bismuth targets with a cyclotron. A clinical trial has been initiated using [^211^At]NaAt for refractory thyroid cancer at Osaka University Hospital (NCT05275946). This trial is expected to be expanded to ^211^At-labeled compounds and antibodies in the near future. [^211^At]GPC1 mAb may be a promising therapeutic as a targeted α-therapy for PDAC and other GPC1-expressing tumors. For clinical application, it is necessary to confirm the safety of GPC1 mAb in humans and ensure its stable supply. We are aiming for clinical companion diagnostic use of [^89^Zr]GPC1 mAb PET ahead of [^211^At]GPC1 mAb in the future clinical trial of the GPC1 mAb drug conjugate.

This study had certain limitations. First, the number of animals in each experiment was limited, and further confirmation of reproducibility is necessary. Second, the biodistribution and potential side effects of [^211^At]GPC1 mAb were not fully evaluated. Future studies should conduct a detailed evaluation, including histologic analysis of the organs at risk, such as the liver and spleen. Third, the chelator-to-antibody ratio of the deferoxamine is still under investigation and should be clarified for future research. Lastly, the tumor xenograft model used in this study exhibited limited stromal formation, which differs from cancer lesions observed in patients. Considering that GPC1 expression is also observed in the stroma of PDAC, it is important to assess the accumulation of GPC1 mAbs labeled with ^89^Zr or ^211^At in patient-derived tumor xenograft models before proceeding to clinical translation.

## CONCLUSION

[^89^Zr]GPC1 mAb PET demonstrated high tumoral uptake in the early phase after administration, indicating its feasibility as a PET probe for detecting GPC1 expression. [^211^At]GPC1 mAb with internalization ability exhibited tumor growth suppression, highlighting the potential of a theranostic approach targeting GPC1. GPC1 mAb holds promise for future applications, from the precise diagnosis of PDAC to GPC1-targeted α-therapy.

## DISCLOSURE

This study was funded by the QiSS program of JST OPERA (grant number JPMJOP1721) and JSPS KAKENHI (grant numbers 16H06278 and 20H05675). Satoshi Serada and Tetsuji Naka declare that a patent for US10077316B2 has been issued and a patent for WO2015098112A1 is pending, which is related to the GPC1 mAb. No other potential conflict of interest relevant to this article was reported.

## Notes

KEY POINTS **QUESTION:** Does GPC1 mAb labeled with ^89^Zr or ^211^At have potential as a theranostic agent? **PERTINENT FINDINGS:** [^89^Zr]GPC1 mAb PET showed high tumoral uptake in the early phase after administration, whereas targeted α-therapy using [^211^At]GPC1 mAb successfully suppressed tumor growth. **IMPLICATIONS FOR PATIENT CARE:** Theranostics using GPC1 mAb with internalization ability labeled with ^89^Zr or ^211^At is a promising approach for the treatment of PDAC.
